# Clinical impact of radiofrequency ablation and stereotactic body radiation therapy for colorectal liver metastasis as local therapies for elderly, vulnerable patients

**DOI:** 10.1002/jgh3.12325

**Published:** 2020-03-20

**Authors:** Naoto Gotohda, Shogo Nomura, Manami Doi, Katsuyuki Karasawa, Takamasa Ohki, Yasuhiro Shimizu, Yoshitaka Inaba, Atsuya Takeda, Haruyuki Takaki, Hiroshi Anai, Masafumi Ikeda, Motokazu Sugimoto, Tetsuo Akimoto

**Affiliations:** ^1^ Department of Hepatobiliary and Pancreatic Surgery National Cancer Center Hospital East Chiba Japan; ^2^ Biostatics Division, Center for Research Administration and Support National Cancer Center Tokyo Japan; ^3^ Department of Surgery Tokyo Metropolitan Cancer and Infectious Diseases Center Komagome Hospital Tokyo Japan; ^4^ Department of Radiation Oncology Tokyo Metropolitan Cancer and Infectious Diseases Center Komagome Hospital Tokyo Japan; ^5^ Department of Gastroenterology Mitsui Memorial Hospital Tokyo Japan; ^6^ Department of Gastroenterological Surgery Aichi Cancer Center Hospital Nagoya Japan; ^7^ Department of Diagnostic and Interventional Radiology Aichi Cancer Center Hospital Nagoya Japan; ^8^ Radiation Oncology Center Ofuna Chuo Hospital Kamakura Japan; ^9^ Department of Radiology Hyogo College of Medicine Nishinomiya Japan; ^10^ Department of Radiology Nara City Hospital Nara Japan; ^11^ Department of Hepatobiliary and Pancreatic Oncology National Cancer Center Hospital East Kashiwa Japan; ^12^ Department of Radiation Oncology National Cancer Center Hospital East Kashiwa Japan

**Keywords:** colorectal liver metastases, elderly patients, radiofrequency ablation, stereotactic body radiation therapy, vulnerable patients

## Abstract

**Background and Aim:**

Surgical resection is the standard local therapy for patients with colorectal liver metastases (CRLM). However, elderly and vulnerable patients sometimes have various organ dysfunctions. We have to conduct nonsurgical local therapies for those patients who might not tolerate surgery or systemic chemotherapy.

**Methods:**

We retrospectively reviewed medical records of 254 patients who underwent local therapies, including surgery, radiofrequency ablation (RFA), and stereotactic body radiation therapy (SBRT), for CRLM from January 2010 to December 2016, at seven tertiary‐care institutions in Japan. This study was designed to include elderly, vulnerable patients who received local therapy for CRLM. For those undergoing liver resection, only those having one or more points of the Charlson comorbidity index (CCI) were enrolled.

**Results:**

Of the total 169 enrolled patients, 122 patients underwent surgery, 42 RFA, and 5 SBRT as the first local therapy for CRLM. Median overall survival from the first local therapy was 5.9 years for the surgery group, 2.7 years for the RFA group, and 3.8 years for the SBRT group. The proportion of the patients with CCI ≧3 was significantly higher in the group of RFA/SBRT than surgery (*P* < 0.0001). In selected patients with CCI ≧3, there was no difference of the median survival time between the surgery group and the RFA group.

**Conclusions:**

We could have other treatment options to provide nonsurgical local therapies (RFA/SBRT) for elderly, vulnerable CRLM patients who have risks for surgery.

## Graphical abstract

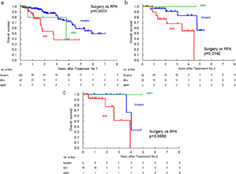

The number of elderly patients with colorectal liver metastasis (CRLM) is increasing. This article indicates the possibility of other treatment options to provide nonsurgical local therapies (radiofrequency ablation/stereotactic body radiation therapy) for elderly, vulnerable CRLM patients who have risks for surgery.

## Introduction

Colorectal cancer (CRC) is the third most commonly diagnosed cancer in males and the second in females, with over 1.2 million newly diagnosed cases and 608 700 deaths estimated to have occurred in 2008.^1^ The liver is the most common metastatic site of CRC. Surgical resection is the standard therapy to cure patients with colorectal liver metastasis (CRLM) if the tumors can be removed completely.

There is a worldwide increase in the elderly population. The number of elderly patients with CRLM is also increasing.^2^ Elderly patients may have various risk factors for surgery under general anesthesia. Liver surgery is a technically demanding operation with high risk of morbidity and even mortality, because of the anatomical complexity with meticulous vascular/biliary system, presence of abundant blood flow, and possibility of massive intraoperative blood loss. Postoperative morbidities may include infectious complications and failure of cardiopulmonary, renal, and/or hepatic function. Thus, for elderly patients having medical comorbidities, surgical indication should be decided carefully.

Systemic chemotherapy can be selected for those elderly CRLM patients with risks for surgical procedures. However, it is sometimes even difficult to provide systemic chemotherapy for elderly patients with interstitial lung disease, renal dysfunction, or hepatic dysfunction. In those patients whose CRLM is localized and without evidence of metastatic CRC to other organs, local therapies including radiofrequency ablation (RFA) or radiation therapy (RT) may play an alternative role to treat CRLM. RFA demonstrated clear survival benefits for patients who were deemed inoperable and/or did not respond to systemic chemotherapy.^3^ Stereotactic body radiation therapy (SBRT) was also shown to be effective for local control in patients with CRLM in a recent study.^4^ Therefore, RFA or SBRT could be used for vulnerable patients with CRLM who were not absolutely suitable for surgical resection or systemic chemotherapy.

It is assumed to be difficult to conduct a randomized clinical trial to test the benefits of these nonsurgical local therapies over surgical resection for those elderly, vulnerable patients with medical comorbidities. Patients with CRLM may require multiple therapeutic modalities as the disease progresses in their clinical course. Therefore, in this study, we collected clinicopathological data of elderly, vulnerable patients with CRLM who underwent surgical or nonsurgical local therapy at multiple institutions, and evaluated their survival outcomes for every opportunity of the local therapy.

## Methods

### *Patients and clinical collection*

**Figure 1:**
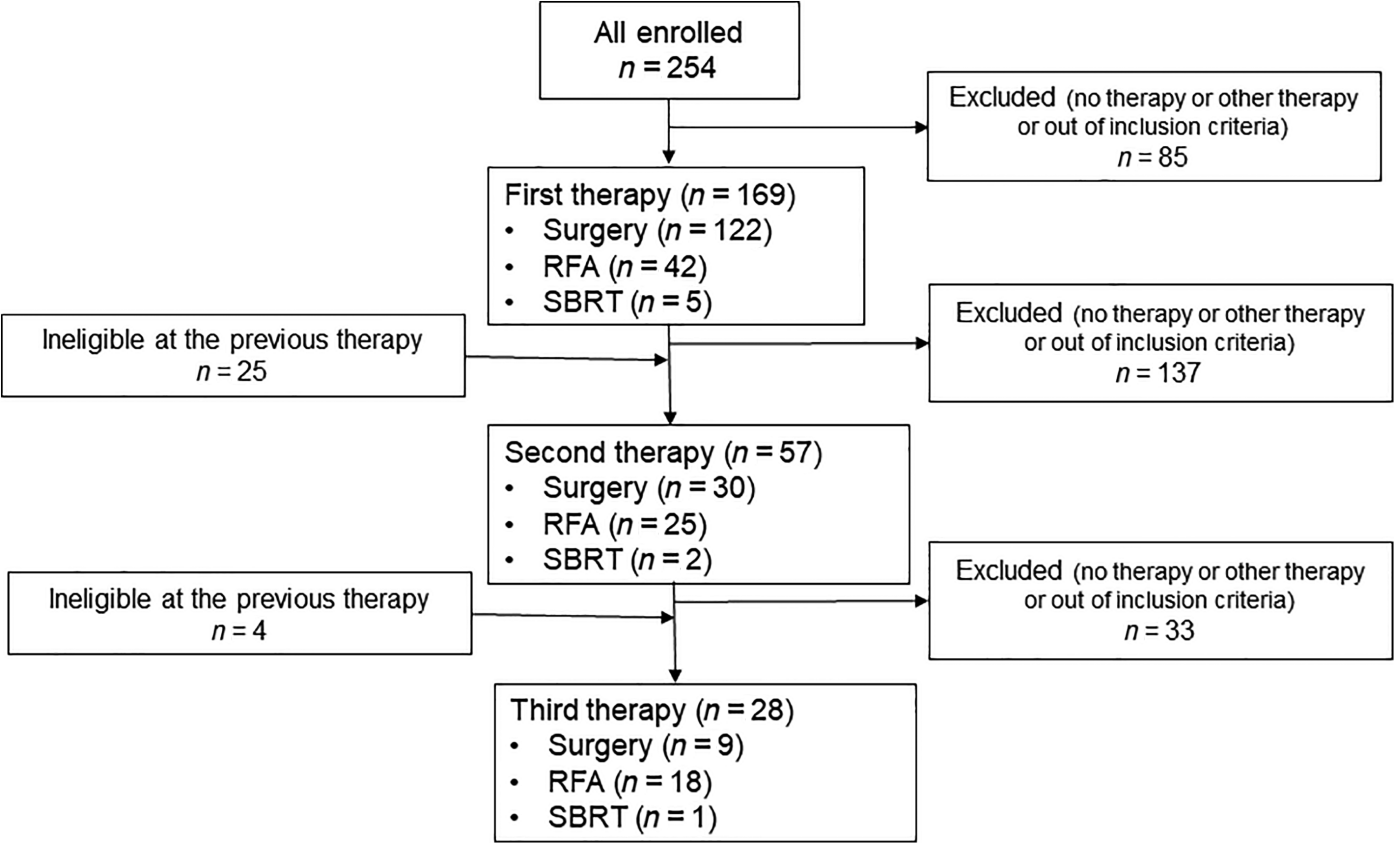
Patient flow diagram. RFA, radiofrequency ablation; SBRT, stereotactic body radiation therapy.

We retrospectively reviewed medical records of 254 patients who underwent local therapies, including liver resection, RFA, and SBRT, for CRLM from January 2010 to December 2016, at seven tertiary‐care institutions in Japan. The study was designed to include elderly, vulnerable patients who received local therapy for CRLM. The inclusion criteria of this study were those patients older than 64 years who underwent local therapy for CRLM with or without systemic chemotherapy. For those undergoing liver resection, only those having one or more points of the Charlson comorbidity index (CCI) were included.^5^ There were a total of 169 patients undergoing local therapy for CRLM meeting these inclusion criteria, while 85 patients were excluded (Fig. 1).

The treatment strategy was similarly decided through multidisciplinary conference at each institution. Liver resection was indicated for CRLM patients usually as the first choice if technically resectable on imaging. Systemic chemotherapy was used at medical oncologist's discretion before and/or after surgery adjuvantly. RFA or SBRT was selectively used for the patients who preferred it to surgery or who were not deemed to have sufficient organ function for surgical resection under general anesthesia or high‐intensity systemic chemotherapy. RFA was performed only with percutaneous approach.

Since there were a considerable number of patients undergoing local therapy for CRLM several times, the enrolled patients were classified based on the timeline of the local therapy. Then, we compared clinicopathological data, posttherapeutic complications, and survival outcomes between the patients who underwent surgical resection and nonsurgical local therapy, including RFA or SBRT.

This study was approved by the Institutional Review Board of each institution and conducted in accordance with the mandates of the Helsinki Declaration.

### *Statistical analysis*

All clinicopathological data of each institution were centralized and analyzed. Categorical variables were evaluated using chi‐square test and are presented as the numbers and percentages. Continuous variables were evaluated using Wilcoxon test and are presented as the median and range. All *P* values were based on two‐sided statistical tests and <0.05 was considered significant. Overall survival was estimated using the Kaplan–Meier method. The starting date of the assessment was the starting day of each local therapy and the ending date was the day of death or recurrence of the case or the day of finishing the follow‐up of the case as outpatient. All calculations were performed using the SAS software (release 9.4) (SAS Institute Inc., Cary, North Carolina, USA) and Microsoft Excel 2013 (Microsoft Corporation, Redmond, Washington, USA).

## Results

### *Local therapies provided for the patients*

Treatment course of the enrolled patients is shown in Figure 1. In total of the 169 patients, 122 patients underwent surgical resection, 42 RFA, and 5 SBRT as the first local therapy for CRLM. Next, 57 patients underwent the second local therapy for recurrence of CRLM: 30 surgical resection, 25 RFA, and 2 SBRT. Then, 28 patients underwent the third local therapy for recurrence of CRLM: 9 surgical resection, 18 RFA, and 1 SBRT.

### *Comparison of clinicopathological characteristics between each treatment timeline*

Clinicopathological characteristics of the enrolled patients according to the treatment timelines are shown in Table 1. The proportion of the patients with CCI ≧3 was significantly higher at the second therapy or third therapy than at the first therapy (*P* = 0.0122 and 0.0021, respectively). Posttherapeutic complications occurred more frequently at the second therapy than the first therapy (*P* = 0.0116).

**Table 1:** Patient characteristics

Variables	First treatment (*n* = 169)	Second treatment (*n* = 57)	Third treatment (*n* = 28)	*P*
Age (median, range)	71 (65–94)	71 (65–90)	72 (65–92)	First *vs* second: 0.9906 Second *vs* Third: 0.4040 third *vs* first: 0.4743
ECOG performance status (0 or 1) (%)	159 (94.1)	56 (98.2)	27 (96.4)	First *vs* second: 0.2066 Second *vs* Third: 0.6035 third *vs* first: 0.6166
Charlson comorbidity index ≧3 (%)	52 (30.8)	28 (49.1)	17 (60.7)	First *vs* second: 0.0122 Second *vs* Third: 0.3143 third *vs* first: 0.0021
BMI (median, range)	22.5 (14.6–33.3)	22.5 (15.1–31.7)	23.6 (16.6–28.3)	First *vs* second: 0.5365 Second *vs* third: 0.4515 Third *vs* first: 0.6347
Child‐Pugh score (B or C) (%)	2 (1.2)	1 (1.8)	0	First *vs* second: 0.7448 Second *vs* third: 0.4848 Third *vs* first: 0.5664
Number of liver metastasis (single) (%)	93 (55.0)	33 (57.9)	18 (64.3)	First *vs* second: 0.6687 Second *vs* third: 0.3589 Third *vs* first: 0.8748
Maximum diameter of liver metastasis (mm) (median, range)	23 (2–120)	22 (5–67)	29.5 (9–60)	First *vs* second: 0.4441 Second *vs* third: 0.0437 Third *vs* first: 0.1044
Other distant metastatic site than liver at the therapy (yes) (%)	29 (17.2)	8 (14.0)	5 (17.9)	First *vs* second: 0.5814 Second *vs* third: 0.6454 Third *vs* first: 0.9279
Uncontrolled other metastatic site than liver (yes) (%)	25 (14.8)	6 (10.5)	3 (10.7)	First *vs* second: 0.4181 Second *vs* third: 0.9789 Third *vs* first: 0.5670
Chemotherapy before or after the therapy (yes) (%)	98 (58.0)	29 (50.9)	14 (50.0)	First *vs* second: 0.3494 Second *vs* third: 0.9394 Third *vs* first: 0.4293
Primary lesion (rectum) (%)	53 (31.4)	18 (31.6)	7 (25.0)	First *vs* second: 0.9755 Second *vs* third: 0.5315 Third *vs* first: 0.4982
Histology (tubular) (%)	163 (96.4)	52 (91.2)	26 (92.9)	First *vs* second: 0.1131 Second *vs* third: 0.7973 Third *vs* first: 0.3724
Lymph node metastasis of primary lesion (n1) (%)	98 (58.0)	35 (61.4)	20 (71.4)	First *vs* second: 0.6505 Second *vs* third: 0.3633 Third *vs* first: 0.1789
Complication after the therapy (yes) (%)	9 (5.3)	9 (15.8)	3 (10.7)	First *vs* second: 0.0116 Second *vs* third: 0.5277 Third *vs* first: 0.2695
Extent of complication (Clavien–Dindo classification grade ≧3A) (%)	6 (3.6)	5 (8.8)	2 (7.1)	First *vs* second: 0.1131 Second *vs* third: 0.7973 Third *vs* first: 0.3724

BMI, body mass index; ECOG, Eastern Cooperative Oncology Group.

### *Comparison of clinicopathological characteristics between local therapies at the first local therapy*

We compared clinicopathological variables of the patients with RFA or SBRT *versus* surgical resection at the time of the first local therapy (Table 2). The proportion of the patients with CCI ≧3 was significantly higher in the group of RFA/SBRT than surgical resection (*P* < 0.0001). The proportion of the presence of other distant metastatic sites than liver and uncontrolled other metastatic sites than liver were also significantly higher in the group of RFA/SBRT than surgical resection (*P* < 0.0001 and *P* = 0.0066, respectively). The proportion of rectal cancer as a primary lesion was significantly lower in the group of RFA/SBRT than surgical resection (*P* < 0.0157). There was no significant difference in the incidence of posttherapeutic complications between RFA/SBRT and surgical resection, but the incidence was zero in the group of RFA/SBRT.

**Table 2:** Comparison of clinicopathological characteristics at the first local therapy

	First treatment for liver metastasis		
Variables	Surgery (*n* = 122)	RFA/SBRT (*n* = 47)	*P*
Age (median, range)	71 (65–85)	72 (65–94)	0.5371
ECOG performance status (0 or 1) (%)	115 (94.3)	44 (93.6)	1.0000
Charlson comorbidity index (CCI) (≧3) (%)	15 (12.3)	37 (78.7)	<0.0001
BMI (median, range)	22.3 (14.6–31.9)	23.5 (15.1–33.3)	0.4212
Child‐Pugh score (B or C) (%)	1 (0.8)	1 (2.1)	0.4610
Number of liver metastasis (single) (%)	66 (54.1)	27 (57.4)	0.8202
Maximum diameter of liver metastasis (mm) (median, range)	23 (2–110)	24 (7–120)	0.8925
Other distant metastatic site than liver at the therapy (yes) (%)	9 (7.4)	20 (42.6)	<0.0001
Uncontrolled other metastatic site than liver (yes) (%)	12 (9.8)	13 (27.7)	0.0066
Chemotherapy before or after the therapy (yes) (%)	66 (54.1)	32 (68.1)	0.1185
Primary lesion (rectum) (%)	45 (36.9)	8 (17.0)	0.0157
Histology (tubular) (%)	118 (96.7)	45 (95.7)	0.6707
Lymph node metastasis of primary lesion (n1) (%)	70 (57.4)	28 (59.6)	0.8628
Complication after the therapy (yes) (%)	9 (7.4)	0	0.0636
Extent of complication (Clavien–Dindo classification grade ≧3A) (%)	6 (4.9)	0	0.1878

BMI, body mass index; CCI, Charlson comorbidity index; ECOG, Eastern Cooperative Oncology Group; RFA, radiofrequency ablation; SBRT, stereotactic body radiation therapy.

### *Survival analyses*

**Figure 2:**
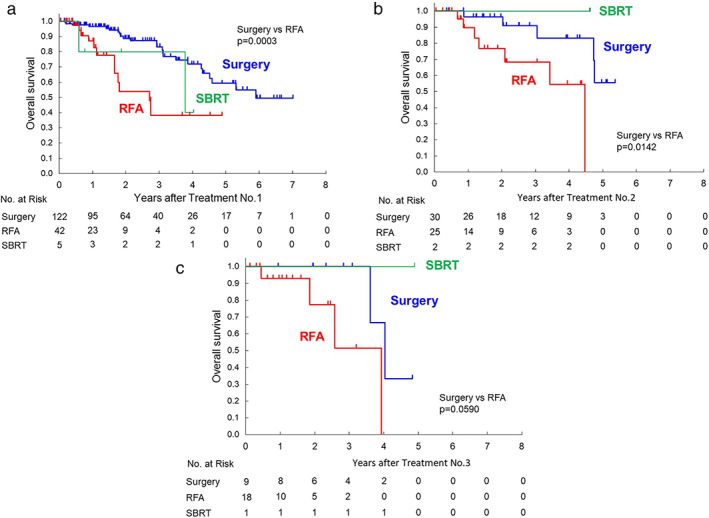
(a) Kaplan–Meier survival curves of 169 colorectal liver metastasis (CRLM) patients undergoing surgery or radiofrequency ablation (RFA) or stereotactic body radiation therapy (SBRT) from the first time of each therapy. (b) Kaplan–Meier survival curves of 57 CRLM patients undergoing surgery or RFA or SBRT from the second time of each therapy. (c) Kaplan–Meier survival curves of 28 CRLM patients undergoing surgery or RFA or SBRT from the third time of each therapy.

**Figure 3:**
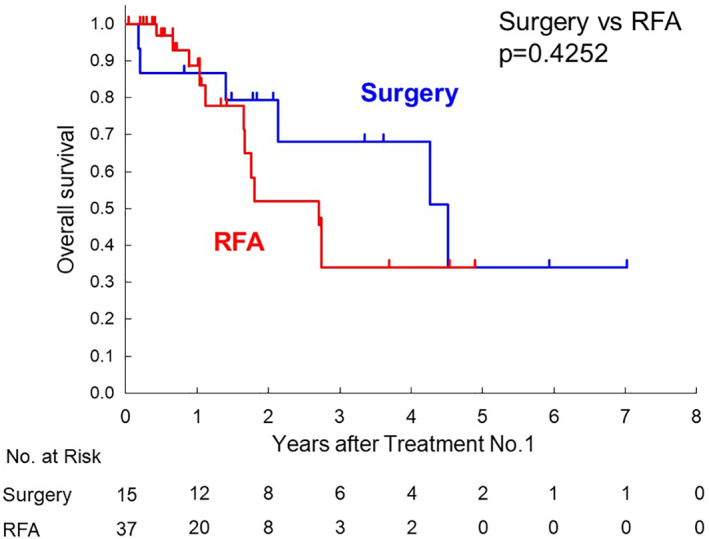
Kaplan–Meier survival curves of 52 colorectal liver metastasis patients with Charlson comorbidity index ≧3 undergoing surgery or radiofrequency ablation (RFA) from the first time of both therapies.

**Figure 4:**
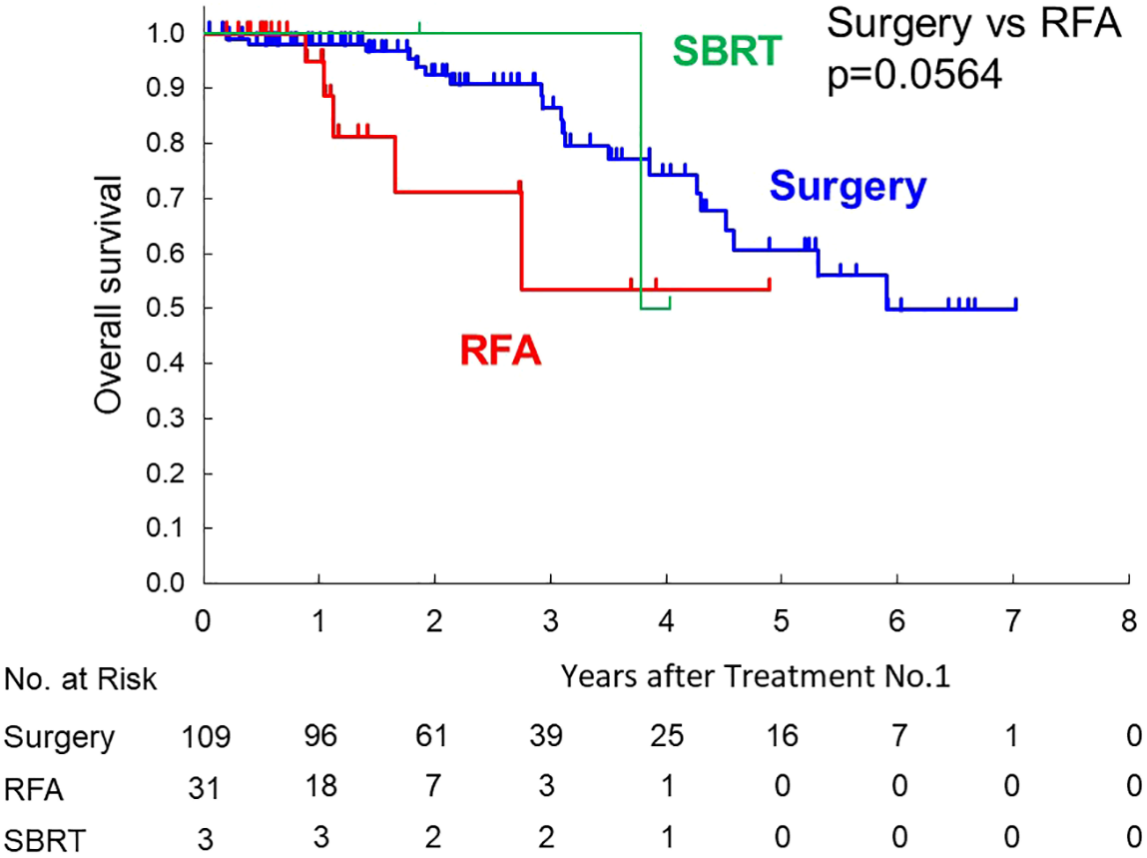
Kaplan–Meier survival curves of 143 colorectal liver metastasis patients undergoing surgery or radiofrequency ablation (RFA) or stereotactic body radiation therapy (SBRT) from the first time of each therapy (excluding patients with other metastases at the therapy).

Median overall survival from the first local therapy was 5.9 years for the surgery group, 2.7 years for the RFA group, and 3.8 years for the SBRT group, as shown in Figure 2a (surgery *vs* RFA: *P* = 0.0003). Survival data from the second or third local therapy are also shown in Figure 2b (surgery *vs* RFA: *P* = 0.0142) or Figure 2c (surgery *vs* RFA: *P* = 0.0590), respectively. In selected patients with CCI ≧3, the median survival time of the surgery group was shorter than all patients with surgery. There was no difference of the median survival time compared with the RFA group (Fig. 3). As shown in Figure 4, in selected patients without other distant metastatic sites than the liver, survival curves for both the RFA group and the surgery group seems to be similar within 1 year after the first therapy.

## Discussion

There were a lot of reports and systematic reviews regarding the outcome after RFA in patients with CRLM.^6^, ^7^, ^8^, ^9^, ^10^, ^11^, ^12^, ^13^, ^14^, ^15^, ^16^ Surgical resection is still the gold standard in the treatment of resectable CRLM.^17^ The National Comprehensive Cancer Network guidelines also provide recommendations of surgical resection or systemic chemotherapy for patients with CRLM.^18^, ^19^ Five‐year survival rates were reportedly 23–66% after surgical resection and 21–49% after RFA.^7^, ^8^, ^9^, ^10^, ^11^, ^12^, ^13^ Those studies demonstrated that survival outcomes were better after surgical resection than RFA for CRLM patients. However, the difference of survival outcomes among the therapeutic modalities could be explained in some points. In the majority of previous studies of which study designs were retrospective, surgical resection was used for those patients with resectable CRLM, while RFA was used for those with unresectable CRLM; therefore, selection bias for local treatment cannot be denied in those studies. In the present study, compared with those patients with surgical resection, those with RFA/SBRT had other distant metastatic sites or uncontrolled other metastatic sites than the liver at the therapy more frequently (Table 2).

Furthermore, the prevalence of comorbidity among patients with CRC reportedly increased from 1995 to 2010.^20^ Aging contributes to more comorbid diseases. Although over 60% of the total incidence of cancer occurs in the elderly (more than 65 years) population, there is a general belief that elderly patients may not be able to tolerate high‐intensity cancer treatments, which may result in this patient population being excluded from prospective clinical trials. The proportion of elderly patients included in clinical trials is significantly lower than the actual proportion of elderly patients suffering from cancer.^21^ The results from clinical trials in a younger generation of patients may not directly be applicable to the treatment for the elderly. Therefore, in clinical situation, it is often difficult to decide the suitable treatment for elderly, vulnerable patients. We need to consider an alternative to the standard therapy especially for elderly, vulnerable patients. As the number of elderly patients with CRLM increases more, the alternative treatment for those patients will be more required in the near future.

Although survival outcomes were better after surgical resection than RFA for CRLM patients, short posttreatment outcomes should also be evaluated for elderly, vulnerable patients. The RFA‐related morbidity was reportedly 4%–8.1%.^22^, ^23^, ^24^, ^25^ In a meta‐analysis comparing the short‐ and long‐term outcomes after RFA and surgical resection,^26^ morbidity was shown to be 9.98% in RFA and 24.1% in surgical resection. Morbidity was higher in the surgical resection group and the relative risk was 2.495 (95% confidence interval, 1.881–3.308). In our study complication occurred in 7.4% after surgical resection but none after RFA/SBRT (Table 2). RFA/SBRT seems less invasive and safer to patients than surgical resection. Therefore, RFA or SBRT can be selected for patients who cannot tolerate surgery due to poor physical condition or inadequate organ function.

RT is also a less invasive local therapy. Recently, SBRT has emerged as a more effective technique of RT than ordinary RT. SBRT is a high‐precision conformal external‐beam radiation technique that ablates targets at extracranial sites by delivering hypofractionated high‐dose radiation while sparing the normal surrounding tissue. SBRT was shown to provide high rates of local control for patients with stage I non‐small cell lung cancer^27^ and 91% of the 3‐year local control rate for patients with hepatocellular carcinoma.^28^ There were some reports showing clinical outcomes after SBRT for patients with metastatic liver tumor. Kirichenko *et al*. reported the local control rate of 93.8% (3/48 failures, 2 for CRLM and 1 for breast cancer liver metastases).^29^ Takeda *et al*. studied the effect of SBRT in patients with hepatic or pulmonary oligometastases from CRC, and reported that the local control rate and overall survival were both 100% at 2 years, especially in 12 patients with CRLM.^4^ In our study, 8 CRLM patients with SBRT developed no local recurrences during the study period.

It is worthwhile evaluating whether elderly patients with organ dysfunction can tolerate high‐intensity therapy or not. The CCI was developed as a scoring system to rank comorbidity into specific risk classification by assigning type and severity scores to a range of specific illnesses.^5^ Although the CCI was not designed to predict perioperative mortality in surgical cohorts, some reports showed the correlation between the CCI and perioperative death in elderly patients. Laor *et al*. analyzed postoperative morbidity and mortality in geriatric patients (≧75 years of age) undergoing general surgery and demonstrated that the score of CCI of early nonsurvivors (death within 30 days after surgery) was significantly higher than that of survivors. The mean CCI score of early nonsurvivors was over 3 points.^30^ In a national cohort study by Doat *et al*., the CCI ≧ 3 was shown to be associated with the risk of death in patients with metastatic CRC. In our study, the proportion of patients with the CCI ≧ 3 having undergone surgical resection was significantly lower than those having other local therapies (12.3% *vs* 78.7%) (Table 2). Surgical candidates should have been selected carefully through preoperative evaluation, especially in elderly, vulnerable patients. Only in patients with CCI ≥3, there was no significant difference in overall survival after surgical resection and RFA in this study (Fig. 3).

Systemic chemotherapy can be considered as another option for patients who are not fit for surgery. However, as systemic chemotherapy can cause adverse events or organ injury, it may be difficult to provide high‐intensity chemotherapy for patients with poor physiological condition. Doat *et al*. reported that elderly patients had a smaller chance of receiving chemotherapy in the advanced setting (48% of patients ≥75 years *vs* 85% of patients <75 years; *P* < 0.0001).^2^ Therefore, noninvasive local therapy can be considered for elderly, vulnerable patients who might not tolerate surgical resection or systemic chemotherapy.

There are some limitations in this study. First, this study is retrospective. The selection bias of the patients for different therapeutic modalities might exist. Second, patients receiving various regimens of systemic chemotherapies were included. Some patients had systemic chemotherapy before and/or after the local therapy. We counted only the number of times of local therapies but not for systemic chemotherapy. We did not analyze the effect of systemic chemotherapy on survival outcomes. Finally, it was difficult to evaluate the survival outcomes after SBRT alone from our data because the number of patients with SBRT was too small. As shown in a few previous reports, it could be expected that SBRT provided good local control for patients with CRLM. Prospective studies to evaluate efficacy of SBRT for CRLM patients should be conducted in the near future.

In conclusion, we could have other treatment options to provide nonsurgical local therapies (RFA/SBRT) for elderly, vulnerable CRLM patients who have risks for surgical resection or systemic chemotherapy and otherwise might be indicated palliative care only.
